# Usefulness of Image Guidance in the Surgical Treatment of Petrous Apex Cholesterol Granuloma

**DOI:** 10.1155/2013/257263

**Published:** 2013-10-22

**Authors:** A. Pietrantonio, G. D'Andrea, I. Famà, L. Volpini, A. Raco, M. Barbara

**Affiliations:** ^1^Department of Neuroscience, Mental Health and Sensory Organs, Division of Neurosurgery, S. Andrew Hospital, University of Rome “Sapienza”, 00189 Rome, Italy; ^2^Department of Neuroscience, Mental Health and Sensory Organs, Division of Otorhinolaryngology, S. Andrew Hospital, University of Rome “Sapienza”, 00189 Rome, Italy

## Abstract

The petrous apex is a pyramid-shaped structure, located medial to the inner ear and the intrapetrous segment of the internal carotid artery. Lesions of the petrous apex can be surgically treated through different surgical routes. Because of the important neurovascular structures located inside the temporal bone, anatomical 3D knowledge is paramount. For this reason, image-guided surgery could represent a useful tool. We report the case of a young woman who came to our observation for a trigeminal neuralgia due to a petrous apex cholesterol granuloma. The lesion was treated through the placement of a drainage tube via an infracochlear approach, with the aid of neuronavigation and intraoperative MRI. Preoperative CT scan images and intraoperative MRI images were fused for surgical planning. The accuracy of the neuronavigation system has proved to be good, and the safety of the procedure was enhanced. Therefore, neuronavigation and intraoperative MRI, though not available in all neurootological centres, should be considered useful tools in these challenging procedures.

## 1. Introduction

The petrous apex is a pyramid-shaped structure that represents the most medial part of the temporal bone [1, 2]. It is located medially to the intrapetrous segment of the internal carotid artery, inner ear, and Eustachian tube [1–3]. Several types of lesions may involve the petrous apex, such as cholesterol granuloma, cholesteatoma, dermoid cyst, mucocele, abscess, primary benign, or malignant neoplasms and metastases [3]. Because of the complex anatomical relationships with the adjacent neurovascular structures (internal carotid artery, jugular bulb, VII and VIII CN, and inner ear), surgery may be highly challenging. In order to respect the aforementioned structures and to achieve a macroscopically total resection of petrous apex lesions, different surgical approaches have been described in the literature. Image-guided surgery could represent a useful tool for surgical planning; therefore [4], we report the case of a young woman who underwent surgical treatment of a petrous apex cholesterol granuloma via an infracochlear approach, with the aid of neuronavigation and intraoperative MRI.

## 2. Case Presentation

**Figure 1:**
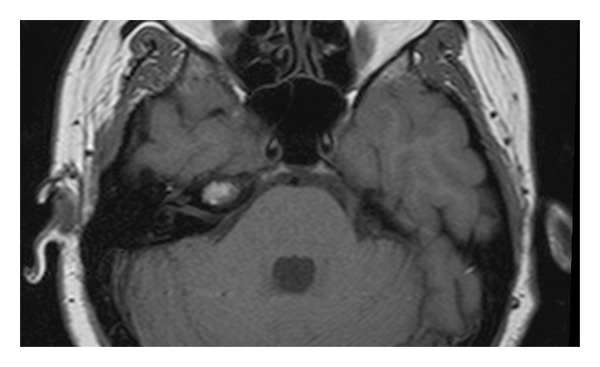
Axial T1-weighted image shows a hyperintense lesion in the right petrous apex.

**Figure 2:**
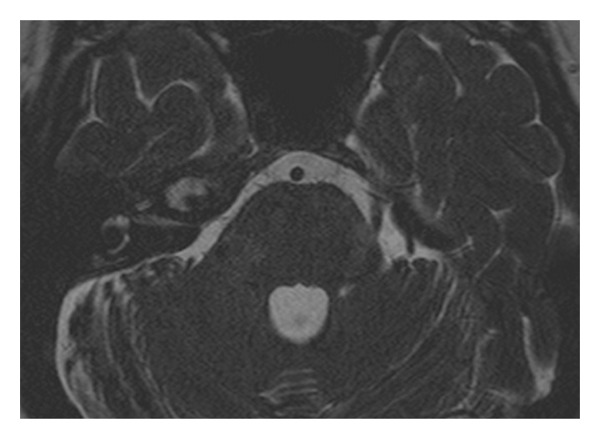
Axial T2-weighted image showes a hyperintense lesion in the right petrous apex.

**Figure 3:**
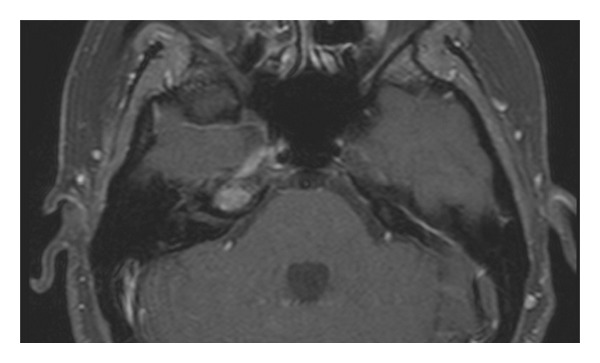
Axial T1-weighted image with Gadolinium showes the absence of postcontrast enhancement in the lesion of the right petrous apex.

**Figure 4:**
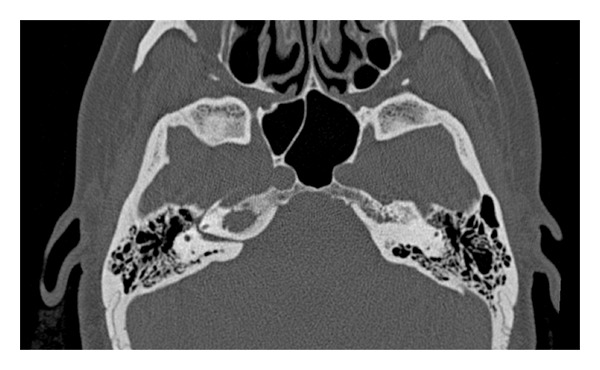
Axial CT scan showes an osteolytic lesion involving the right petrous apex.

**Figure 5:**
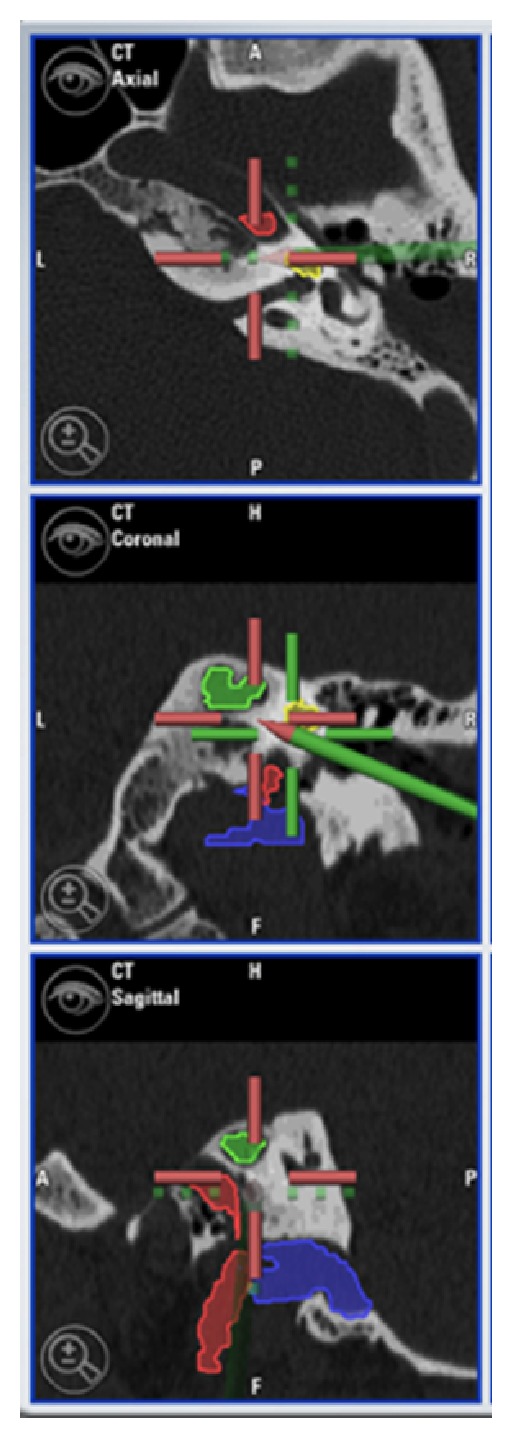
Intraoperative screenshot shows the direction of drilling and the relationship between the surgical route and the surrounding structures (*blue*: jugular bulb; *yellow*: cochlea; *red*: internal carotid artery; *green*: petrous apex lesion).

**Figure 6:**
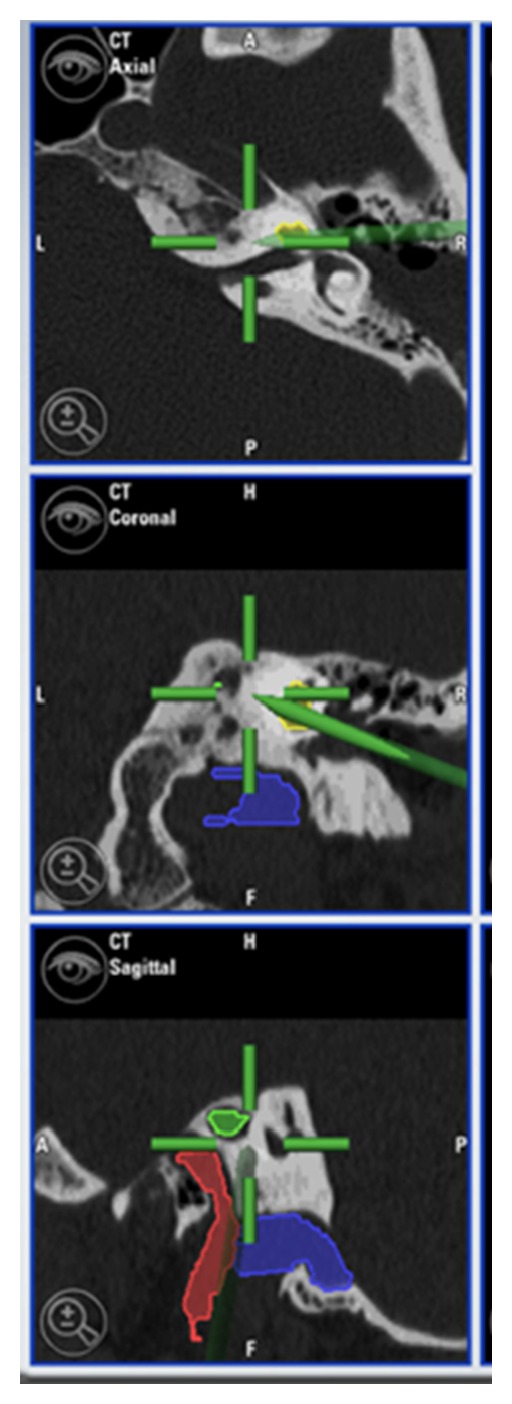
Intraoperative screenshot at the end of the drilling. The petrous apex lesion has been reached without damaging the surrounding structures (*blue*: jugular bulb; *yellow*: cochlea; *red*: internal carotid artery; *green*: petrous apex lesion).

A 36-year-old woman came to our observation because of a three-month history of left trigeminal neuralgia with pain distributed along the ophthalmic division of the fifth cranial nerve. The neurological examination revealed the absence of neurological deficits. MRI with gadolinium showed a hyperintense lesion on both T1- and T2-weighted images, with no postcontrast enhancement (Figures 1, 2, and 3), highly suggestive for a petrous apex cholesterol granuloma [5]. CT scan showed an osteolytic lesion involving the right petrous apex (Figure 4). At the beginning, a wait- and see-strategy was decided, considering the neuroradiological features of the lesion and the fact that patient's symptoms were not highly disabling. As the lesion started growing (2 mm in A-P diameter at two years of followup) and the patient's symptoms worsened despite analgesic therapy, the surgical treatment was indicated. Pre- and postoperative audiometry showed normal function. With the aid of neuronavigation and intraoperative MRI (1,5-T magnet, Magnetom Sonata, Siemens AG, Medical Solutions, Erlangen, Germany; T1-weighted sequences before and after administration of paramagnetic contrast medium, T2-weighted sequences, FLAIR sequences, and angiographic sequences) (Figures 5 and 6), a transcanal infracochlear approach was performed, and a drainage tube was placed inside the cystic cavity. Neurophysiological monitoring of the VII CN was performed. Preoperative CT scan images and intraoperative MRI images (slice thickness of CT and MRI images: 1 mm) were matched on the neuronavigation system (Planning software iPlan 2.6, BrainLab AG, Feldkirchen, Germany). The histological examination revealed a cholesterol granuloma. In the early postoperative period, the patient showed an improvement of the preoperative symptoms. Followup of MRI with Gad and CT-scan at 6 months showed a reduction of the volume of the lesion and a stable aeration of the cystic cavity. The patient is symptom-free one year after surgery.

## 3. Discussion

The petrous apex can be anatomically described as a pyramid-shaped structure that can be divided in a posterior and an anterior part by a line passing in the coronal plane through the internal auditory canal [2]. The base of the pyramid is represented by the otic capsule and the inferior surface by the jugular fossa and the inferior petrosal sinus; the superior surface (meatal plane) extends from the arcuate eminence to Meckel's cave, and the posterior surface is located in front of the cerebellopontine angle. The petrous apex is usually made up of bone marrow or dense bone, and only in 9% to 30% of subjects some degree of pneumatization can be seen [3]. Due to its deep location and to the surrounding anatomical structures (internal carotid artery, VII and VIII CN, and cochlea), the access to this region could be challenging. Several surgical approaches have been described in the literature, passing through the middle fossa, the labyrinth, or along lines of air cells tracts. These different surgical approaches have been developed considering the position, the dimension, and the histopathological nature of the lesion to be treated. While in certain types of lesions the goal of treatment is complete surgical removal and a wide exposure of the petrous apex is required, in other lesions (i.e., cholesterol granuloma) the permanent drainage of the surgical cavity could represent a viable and effective option and can be performed as a minimally invasive procedure. The infracochlear and the infralabyrinthine approaches are defined as approaches that follow the air cells tracts below the labyrinth. The first one was described by Giddings et al. for the treatment of cholesterol granulomas of the petrous apex [6]. In this approach, a tympanomeatal flap, attached to the umbo, is elevated superiorly, and the chorda tympani represents the posterior border of the dissection that will proceed in the hypotympanum between the inferior border of the basal turn of the cochlea superiorly, the internal carotid artery anteriorly, and the jugular bulb inferiorly [6, 7]. If compared to the infralabyrinthine approach, the infracochlear approach provides a more direct, shorter, but surely narrower route to reach the petrous apex [8, 9]. On the other hand, Leung et al. demonstrated that temporal bones with petrous apex pneumatization have an overall larger infracochlear space (mean diameter 5.7 mm) than sclerotic petrous apex (mean diameter 5.1 mm); however, both of them provide adequate space for an infracochlear exposure [10]. The infralabyrinthine approach allows a wider exposure of the petrous apex and does not require surgical manipulation on the eardrum and exposure of the internal carotid artery, although the dissection can be impeded by a high jugular bulb, even after performing a wide mastoidectomy [6, 8, 11]. The choice of either approach is mainly based on the position of the jugular bulb. However, surgeon's experience and skill play an important role, considering the complex anatomy of this region. In this patient, the preoperative neuroimaging suggests a probable diagnosis of cholesterol granuloma and the infracochlear approach with placement of a drainage tube was chosen. Other surgical approaches have been described in the literature for the treatment of a petrous apex lesion [9, 11–15]; the translabyrinthine approach provides the most direct route to the petrous apex, but it should be reserved for patients with no serviceable hearing, considering the unavoidable labyrinthine damage. The middle fossa approach allows a complete removal of the pseudocapsule; however, a stable aeration of the cavity cannot be maintained, and a craniotomy with temporal lobe retraction must be performed [11, 12]. The transsphenoidal endoscopic approach has been proposed only when the cystic wall comes in contact with the sphenoid sinus walls, but the possibility of a wide erosion of the carotid canal and the dural involvement make this procedure challenging and hazardous [11, 12]. Hence, the infralabyrinthine and infracochlear routes are the most viable options for the surgical drainage of a cholesterol granuloma or when a biopsy should be performed in patients with serviceable hearing. The limited exposure obtained with these approaches frequently does not allow radical removal of petrous apex lesions different from cholesterol granulomas, while the placement of a drainage tube for the maintenance of a permanent cavity ventilation is feasible. In fact, the goal of surgery of a cholesterol granulomas should not be the complete removal of the pseudocapsule but the maintenance of a stable ventilated cavity [11]. Surgery of petrous apex lesions requires a detailed and 3-dimensional knowledge of the anatomy of the temporal bone. Hence, image guidance could represent a useful tool when the drilling of the petrous bone must be performed, in order to reduce the surgical-related damage to the adjacent neurovascular structures [4, 10]. Moreover, image-guided surgery in petrous bone lesions has proved to be highly accurate due to the absence of intraoperative shifting [4]. In our experience, the preoperative CT scan with 1 mm slice thickness was combined with an intraoperative MRI for neuronavigation (T1-weighted with and without Gad, T2-weighted, FLAIR, and angiographic sequences). Skin fiducials were not used preoperatively, because preoperative CT scan images and intraoperative MRI images could be easily matched on the neuronavigation system. While CT scan provided a higher quality representation of the petrous bone, especially for the position of the basal turn of the cochlea (upper limit of drilling), the fusion of MRI and CT images was useful in delineating the area between the ICA (anterior limit of drilling) and the jugular bulb (inferior limit of drilling). Image acquisition and preoperative planning took 15 minutes, and the neuronavigation system showed a good accuracy; thus, operative time was not highly prolonged, and the safety of the procedure was consistently enhanced. 

In conclusion, this report outlines the usefulness of intraoperative image guidance for transtemporal approaches to the petrous apex, especially in patients with serviceable hearing and no preoperative cranial nerve deficits. The absence of brain shifting and the advancements in neuroimaging techniques make it highly accurate, without increasing surgical time. Moreover, our personal experience emphasizes the advantage of matching preoperative CT and intraoperative MRI scans, since the different type of information provided by these two studies enhance the accuracy of the 3D representation of the temporal bone anatomy, allowing a safer drilling of the petrous bone.
